# Supplementary material for: A framework for community curation of interspecies interactions literature
Source: eLife. 2023 Jul 4;12:e84658. doi: 10.7554/eLife.84658 (PMC10319440; doi:10.7554/eLife.84658)
Supplement: Supplementary file 1. [file elife-84658-supp1.docx]

# **Supplementary file 1**. Mapping display name to relation name for Annotation Extensions in PHI-Canto.

| **PHI-Canto display name** | **Annotation extension relation** |
| --- | --- |
| compared to control | compared_to_control |
| gene-for-gene interaction | gene_for_gene_interaction |
| inverse gene-for-gene interaction | inverse gene_for_gene_interaction |
| penetrance | has_penetrance |
| extent of infectivity | infective_ability |
| host tissue infected | infects_tissue |
| outcome of interaction | interaction_outcome |
| observed in organ | observed_organ |
| affected proteins (add TWO i.e., both binding partners) | assayed_using |
| assayed protein | assayed_using |
| assayed RNA | assayed_using |
| severity | has_severity |
|  |  |
