# Supplementary material for: A framework for community curation of interspecies interactions literature
Source: eLife. 2023 Jul 4;12:e84658. doi: 10.7554/eLife.84658 (PMC10319440; doi:10.7554/eLife.84658)
Supplement: Supplementary file 3. [file elife-84658-supp3.docx]

# **Supplementary file 3.** PHI-base nine high level term mapping to PHI-Canto.

| **PHI-Canto term name^1^** | **Definition** | **Term ID** | **High level term annotated to …** |
| --- | --- | --- | --- |
| Increased resistance to chemical^2^ | A single species population phenotype in which a population shows increased resistance to a chemical stimulus.^3^ | PHIPO:0000022^5^ | Genotype^7^ with PHIPO term |
| Increased sensitivity to chemical^2^ | A single species population phenotype in which a population shows decreased resistance to a chemical stimulus.^4^ | PHIPO:0000021 | Genotype with PHIPO term |
| Inviable population | An organism population phenotype in which no organisms in the population are viable. | PHIPO:0000513 | Genotype with PHIPO term |
| Loss of pathogenicity | A phenotype where the ability of a pathogen, to produce an infectious disease in another organism is abolished (pathogenicity was present and is now absent). | PHIPO:0000010 | Metagenotype^8^ with AE^9^ extent of infectivity |
| Unaffected pathogenicity | A phenotype where the ability of a pathogen, to produce an infectious disease in another organism is unaffected (i.e., the same as wild type, it could be pathogenic or non-pathogenic). | PHIPO:0000004 | Metagenotype with AE extent of infectivity |
| Reduced virulence | A phenotype where the degree to which a pathogen (species or strain) is able to cause infectious disease in another organism is increased (i.e., more symptoms than normal). | PHIPO:0000015 | Metagenotype with AE extent of infectivity |
| Increased virulence | A phenotype where the degree to which a pathogen (species or strain) is able to cause infectious disease in another organism is increased (i.e., more symptoms than normal). | PHIPO:0000014 | Metagenotype with AE extent of infectivity |
| Loss of mutualism | A phenotype in which the balance of symbiotic mutualism has been disrupted compared to the normal interaction and the endosymbiont organism is able to show greater biomass within the host and/or the formation of visible disease formation symptoms compared to the normal interaction. | PHIPO:0000207 | Metagenotype with AE extent of infectivity |
| Effector-mediated modulation of host process by symbiont | A process mediated by a molecule secreted by a symbiont that results in the modulation (either activation or suppression) of a host structure or process. The host is defined as the larger of the organisms involved in a symbiotic interaction. | GO:0140418^6^ | Gene^10^ with GO Biological Process term (or descendants)^11^ |

^1^ Some term names have been updated since initial publication in Urban et al., 2015 NAR (PMID:25414340). Specifically, ‘inviable population’ was formerly ‘lethal’ and ’loss of mutualism’ was formerly ‘enhanced antagonism’.

^2^ Lower ranked terms, containing specific chemicals names, are mapped up to this term.

^3^ Additional definition gloss: Resistance to a chemical is usually measured by determining the maximum concentration of the chemical at which a population grows and divides.

^4^ Additional definition gloss: Resistance to a chemical is usually measured by determining the maximum concentration of the chemical at which a population grows and divides. Typically, populations are deemed sensitive to a chemical if they stop growing (and may die) at a concentration of the chemical that allows wild type populations to grow.

^5^ PHIPO is the Pathogen-Host Interaction Phenotype Ontology.

^6^ GO is the Gene Ontology.

^7^ A single species genotype can be either a pathogen or a host genotype.

^8^ A metagenotype consists of both a pathogen genotype and a host genotype in the context of a pathogen-host interaction.

^9^ AE is an Annotation Extension. Annotation extensions enable additional data to be related to a primary annotation.

^10^ This curation type does not refer to annotating a phenotype to a genotype, but instead refers to annotating a GO term to a gene product.

^11^ The curator has the option to add a GO Molecular Function term annotation to the pathogen effector, if known.
